# Integrated regulation of chondrogenic differentiation in mesenchymal stem cells and differentiation of cancer cells

**DOI:** 10.1186/s12935-022-02598-8

**Published:** 2022-04-29

**Authors:** Xiaohui Yang, Shifeng Tian, Linlin Fan, Rui Niu, Man Yan, Shuo Chen, Minying Zheng, Shiwu Zhang

**Affiliations:** 1 Nankai University School of Medicine, Nankai University, Tianjin, 300071 People’s Republic of China; 2 Graduate School, Tianjin Medical University, Tianjin, 300070 People’s Republic of China; 3 Department of Pathology, Graduate School, Tianjin University of Traditional Chinese Medicine, Tianjin, 301617 People’s Republic of China; 4 Department of Colorectal Surgery, Tianjin Union Medical Center, Tianjin, People’s Republic of China; 5 Department of Pathology, Tianjin Union Medical Center, Tianjin, 300071 People’s Republic of China

**Keywords:** Chondrogenic differentiation, Mesenchymal stem cells, Signaling pathway, Non-coding RNA, Cancer stem cell, Polyploidy giant cancer cells

## Abstract

Chondrogenesis is the formation of chondrocytes and cartilage tissues and starts with mesenchymal stem cell (MSC) recruitment and migration, condensation of progenitors, chondrocyte differentiation, and maturation. The chondrogenic differentiation of MSCs depends on co-regulation of many exogenous and endogenous factors including specific microenvironmental signals, non-coding RNAs, physical factors existed in culture condition, etc. Cancer stem cells (CSCs) exhibit self-renewal capacity, pluripotency and cellular plasticity, which have the potential to differentiate into post-mitotic and benign cells. Accumulating evidence has shown that CSCs can be induced to differentiate into various benign cells including adipocytes, fibrocytes, osteoblast, and so on. Retinoic acid has been widely used in the treatment of acute promyelocytic leukemia. Previous study confirmed that polyploid giant cancer cells, a type of cancer stem-like cells, could differentiate into adipocytes, osteocytes, and chondrocytes. In this review, we will summarize signaling pathways and cytokines in chondrogenic differentiation of MSCs. Understanding the molecular mechanism of chondrogenic differentiation of CSCs and cancer cells may provide new strategies for cancer treatment.

## Introduction

The cartilage is a connective tissue composed of chondrocytes and their surrounding matrix, which mainly contains collagen type II and proteoglycans. Chondrogenic differentiation, the formation of chondrocytes and cartilage tissues, originates from the migration and condensation of mesenchymal stem cells (MSCs) [1]. Next, chondroprogenitor cells form, proliferate, and differentiate into chondrocytes [2]. Chondrocytes end up as resting cells to form the articular cartilage or undergo proliferation, hypertrophy, and apoptosis in a process termed endochondral ossification, thereby replacing the hypertrophic cartilage with bone [3]. Neural crest cells of the neural ectoderm, sclerotome of the paraxial mesoderm and somatopleure of the lateral plate mesoderm, which give rise to craniofacial bones, axial skeleton and skeleton of the limbs respectively, are the main source of mesenchymal stem cells [4]. Currently, MSCs are reported to be isolated and cultured from a wide range of tissues including adipose tissue [5], bone marrow [6], synovial membrane [7] and fetal appendages, such as the amniotic membrane, umbilical cord, and chorionic plate [8–10]. Because of their variable source and easy availability, MSCs have been widely used in cartilage tissue engineering [11]. Not only MSCs, but cancer stem cells (CSCs), a group of quiescent cell types that can drive cancer growth and reconstruct their heterogeneity, can differentiate into mesenchymal phenotypes, such as prostate cancer cell lines or melanoma cancer stem cells into osteocytes and adipocytes [12, 13]. In this review, we discuss the regulatory mechanism of MSCs differentiating into chondrogenic lineages, including signaling pathways, key proteins as well as other factors which have been shown to have an important role in chondrogenic differentiation.

## Signaling pathways of chondrogenesis in mesenchymal stem cells

**Fig. 1:**
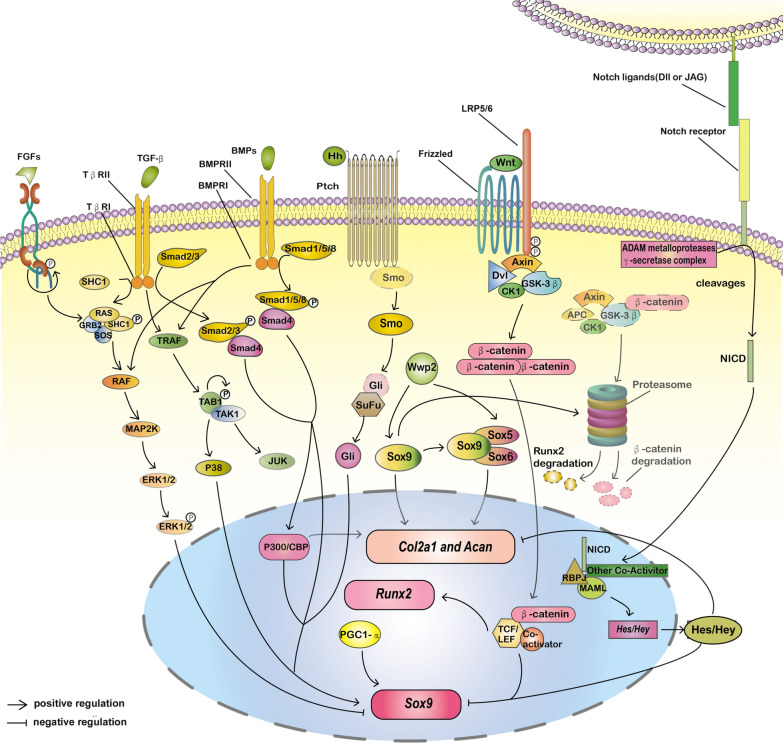
Signaling pathways and proteins involved in chondrogenic differentiation of mesenchymal stem cells. R-SMAD-dependent TGF-β and Hedgehog pathways can promote the overexpression of Sox9, whereas Notch and Wnt pathways can inhibit chondrogenic transcription factor Sox9. FGF can act on R-SMAD-independent TGF-β pathway, thus playing a part in chondrogenic differentiation. There are other molecules that interact with Sox9, such as osteogenic transcription factor Runx2, peroxisome proliferator-activated receptor-gamma coactivator 1-alpha (PGC1-α) and the member of histone acetyltransferase family P300/CBP

The proliferation and differentiation of mesenchymal cells to chondrocytes, or chondrogenic differentiation, are a complex process regulated by multiple elements, which contain intracellular proteins, receptor ligands, and transcription factors, and disruption in signaling can result in defective chondrocyte production. Signaling pathways involved in lineage determination of MSCs towards chondrogenesis is showed in Fig. 1.

### TGF-β pathway exerts positive effects on chondrogenic differentiation

According to the interaction between distinct receptors and the ligands on the membrane, TGF-β family is divided into the TGF-β (transforming growth factor-β)/activin/nodal subfamily and BMP (bone morphogenetic protein)/growth differentiation factor (GDF)/anti-Mullerian hormone subfamily [14]. According to the participation of protein SMAD, intracellular pathways of TGF-β are separated into receptor SMAD (R-SMAD)-dependent and R-SMAD-independent [15]. The former is phosphorylated by type I kinase, thereby activating specific SMAD proteins complex that translocate into the nucleus to regulate the activation of target genes, such as chondrogenic transcription factor Sox9 [16, 17]. The latter includes mitogen-activated protein kinase (MAPK) pathways involving TGF-β-activated kinase 1 (TAK1) or extracellular signal-regulated kinase 1 and 2 [15]. The three ligands (TGF-β1, TGF-β2, and TGF-β3) have ability to induce chondrogenic differentiation, all of which bind to TGF-β type II receptor and TGF-β type I receptor and activate intercellular protein SMAD 2 and 3 [18]. TGF-βs stimulates the expression of cartilage-specific extracellular matrix proteins such as type II collagen and aggrecan [19, 20]. In a micro-pellet model, a single-day treatment of TGF-β1 was a sufficiency of stimulating bone marrow stromal cells (BMSCs) differentiation towards cartilage [21]. Amniotic MSCs overexpressing TGF-β1 expressed cartilage-specific genes and showed intense Safranin O and Alcian blue staining [22]. For cartilage tissue engineering, TGF-β1 is widely used to induce chondrogenic differentiation in MSCs [23, 24], but several studies have stated that TGF-β2 and TGF-β3 are more efficient for the chondrogenic induction with higher production of collagen II and aggrecan and glycosaminoglycan deposition [25, 26].

BMPs recruit MSCs, promote condensation or proliferation, and subsequently trigger their differentiation [27]. BMPs transduce signals through the formation of heteromeric complexes of BMP types II and I receptors and phosphorylation of intracellular protein SMAD 1, 5 and 8. Among the BMP isoforms, the most widely studied are BMP-2, BMP-3 (osteogenin), BMP-4, BMP-6, BMP-7 (also known as osteogenic protein-1), and BMP-9 [18, 28]. BMP-2/4 enhance the recruitment of mesenchymal precursors for cartilage condensations, and regulate the condensation size [29]. Human muscle-derived stem cells (hMDSCs) transfected with lenti-BMP2/GFP vector could enhance the capacity of hMDSCs to differentiate into cartilage, as confirmed by Alcian blue and Col2a1 positive staining [30]. BMP4, BMP11, BMP6, BMP7, BMP9, BMP13 and BMP14 have been reported to up-regulate chondrogenic markers Sox9, Sox5, and Sox6 [31].

In terms of R-SMAD-independent pathway, deletion of Tak1 in limb mesenchyme cells could result in the inactivation of the downstream MAPK target p38, as well as impaired the activation of the BMP/SMAD signaling pathway, which the differentiation of the chondrocyte lineage was interrupted [32]. Noggin subordinated with BMP signaling inhibitors and could be induced by TGF-β1 during the recruitment of progenitor cells into cartilage elements [33]. ERK1/2 is activated upon TGF-β1 stimulation or BMP2 administration and acts as the passive modulator of chondrogenic differentiation [34, 35], but this inhibition of chondrogenic differentiation was covered by the positive effect of R-SMAD-dependent pathway.

### Hedgehog (Hh) pathway plays a relevant role in chondrogenic differentiation

**Fig. 2:**
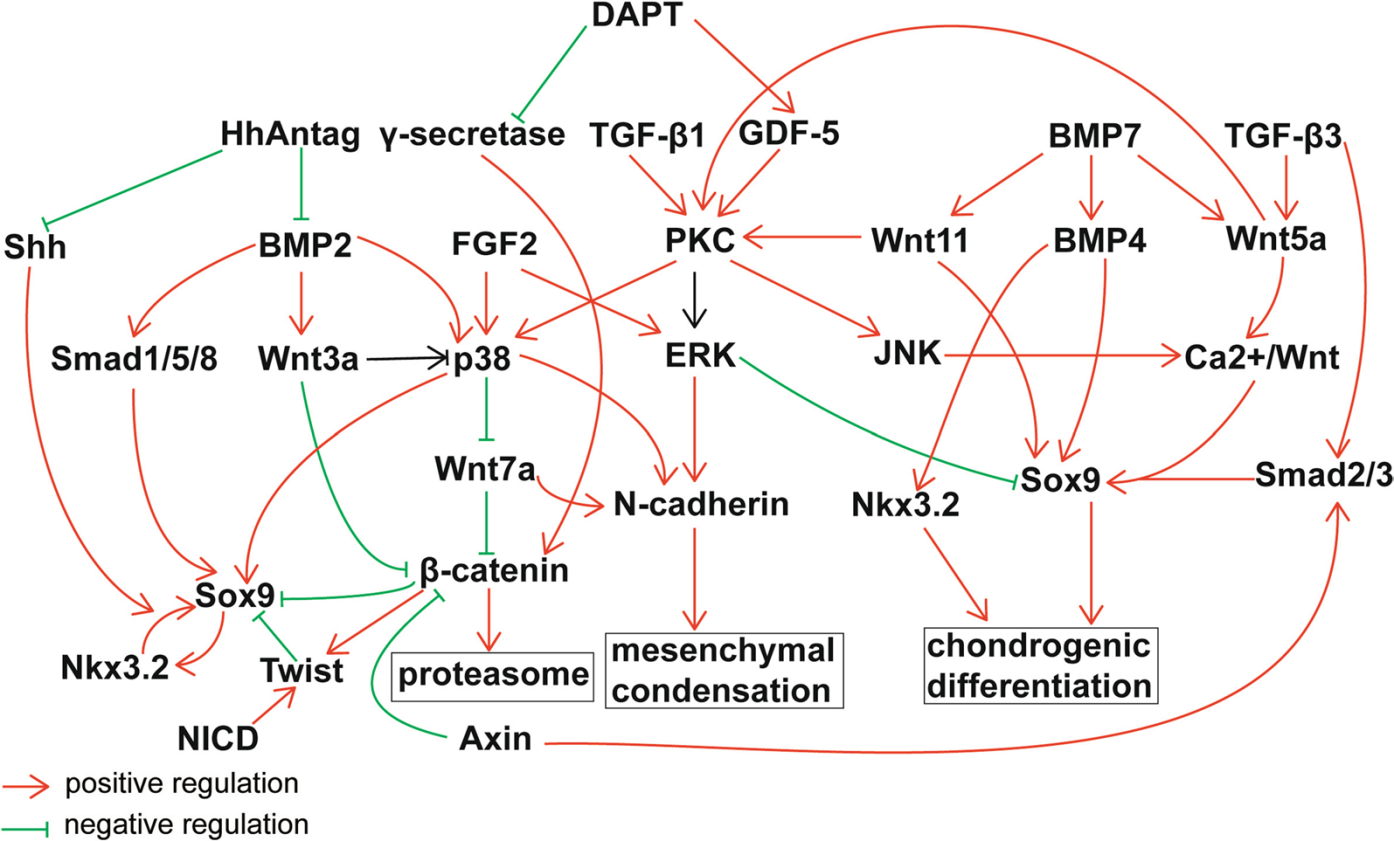
Integrated regulation of chondrogenesis differentiation from mesenchymal stem cells. Red arrows indicate promoting effect and green arrows with lines indicate inhibiting effect

Hh was first identified in Drosophila body plan [36]. Smoothened (Smo) is a receptor, which possesses an ability of activating intracellular signals repressed by Patched (Ptch) [37]. When secreted by the sending cell, Hh ligands bind to Ptch on the receiving cell, and this action can relieve the negative effect of Ptch on Smo, which initiates Hh signal transmission. In mammals, zinc finger proteins Gli1, Gli2, and Gli3 are involved in the transcriptional regulation [38], and sonic hedgehog (Shh), desert hedgehog (Dhh), and Indian hedgehog (Ihh) are ligands in Hh pathway [39]. Dhh is required for spermatogenesis and formation of neuronal sheaths [40], but rarely been reported in chondrogenic differentiation. There is the high-level expression of Ihh in proliferating limb bud mesodermal cells, which subsequently differentiate into osteo-chondroprogenitor after mesenchymal migration and condensation [41]. Compared to TGF-β1 and BMP-2, Ihh is also a potent inducer of chondrogenic differentiation of primary MSCs [42]. Shh takes part in morphogenesis of the muscle, hair, teeth, lung and gut, patterning of body limbs and cell fates of neural progenitors [43]. BMSCs transfected with Shh induced chondrogenic differentiation in the rotary cell culture system [44]. Exogenous Shh could induce the expression of the transcription factor Sox9 in the somatic tissue, and upregulated Sox9 expression level could induce robust chondrogenesis via BMP signals [45]. The Hh signaling antagonist HhAntag could modulate the BMP2-mediated the canonical SMAD1/5/8 and non-canonical p38/MAPK signaling [46] (Fig. 2).

### Notch pathway negatively regulates chondrogenic differentiation

Notch signaling works via cell–cell contact, where transmembrane ligands on one cell recognize transmembrane receptors on its adjacent cells. This interaction can activate intracellular proteases, such as ADAM metalloprotease and γ-secretase complex, thereby contribute to proteolytic cleavage of the receptor [47]. After cleavage, notch intracellular domain (NICD), the COOH-terminal portion of the receptor, is released and translocated to the nucleus to form a complex with the transcription factor CSL (also known as RBP-JK) and the transcriptional coactivator mastermind-like 1 [48]. The ultimate complex targets the hairy and enhancer of split (Hes) and Hes-related with YRPW motif (Hey) [49]. Unlike TGF-β and BMP pathways, Notch signaling inhibits chondrogenic differentiation via interactions between four receptors (Notch1–5) and at least five ligands including two members of the Jagged (JAG) family and three members of the delta-like family (Dll) [48, 50]. This suppression regulates the expression of the chondrogenic transcription factor Sox9, whereas it is irrelevant to the regulation of cartilage matrix catabolism [51, 52]. Cartilage regeneration induced by placenta-derived mesenchymal stromal cell (PMSC) could be promoted by inhibiting the Jagged1 (JAG1) peptides of Notch pathway [53, 54]. Notch inhibition of chondrogenic differentiation is regulated by the transcription factor Twist1, which cooperates with a putative NICD/RBPjK binding element in the promoter region [55]. Overexpression of ligand Dll2 could strongly promote the activation of p38/MAPK rather than extracellular signal-regulated kinase 1/2 and c-Jun N-terminal kinase, thereby inhibiting the chondrogenic differentiation of ATDC5 (a kind of cartilage cell line) [56].

### Wnt pathway influences chondrogenic differentiation

Wnt pathway can be grouped into two categories based on the participation of β-catenin in signal transduction. One is canonical (β-catenin-dependent), and the other is non-canonical (β-catenin-independent) [57]. With respect to the β-catenin-dependent pathway, Wnt proteins bind a heterodimeric receptor complex, comprised of a Frizzled (Fzd) and an LRP5/6 protein [58]. This binding inhibits GSK-3β phosphorylation to β-catenin and leads to the stabilization of β-catenin. β-catenin in stable state accumulates in the nucleus and binds T cell-specific factor/lymphoid enhancer-binding factor (TCF/LEF), resulting in activation of target genes [59, 60]. In the deficiency of Wnt ligands, the cytoplasmic complex consisting of the scaffolding protein Axin, adenomatous polyposis coli, casein kinase 1, and GSK-3β can phosphorylate β-catenin, resulting in its ubiquitination and subsequent proteasomal degradation [61]. The non-canonical pathway is split into two branches, the Wnt/calcium pathway and the planar cell polarity [62]. β-catenin-mediated canonical Wnt signaling inhibits chondrogenic differentiation, while non-canonical pathways promote this differentiation [18, 63]. As the member of canonical Wnt pathway, Wnt3a plays a dual role on chondrogenic capacity of MSCs [64–66]. The non-canonical factor Wnt5a augments cartilage formation, collagen fiber rearrangement, and remarkably enhances glycosaminoglycan and collagen deposited in vivo [67]. The synergistic effect of Wnt5a and TGF-β3 stimulated the activation of p38/MAPK pathway, a positive regulator of chondrogenic differentiation [68]. TGF-β1-mediated MAP kinase activation could promote the accumulation of intracellular β-catenin, and increase the expression of Wnt7a and N-cadherin (mesenchymal condensation marker) [69].

Wnt inhibitors and activators also play a significant role in cartilage homeostasis and development. As a Wnt antagonist, FRZB blocked canonical Wnt signaling and increased the production and deposition of glycosaminoglycan with overexpression of chondrogenic markers (Sox9 and Col2a1) [70]. Lithium chloride and CHIR99021 (CHIR), commercially available Wnt agonists, not only stimulate MSC proliferation, but also enhance the chondrogenic capacity of MSCs [71].

### Fibroblast growth factor (FGF) signaling pathway is associated with chondrogenic differentiation

FGF signaling pathway involves in many physiological processes comprising digit morphogenesis, limb organogenesis, cerebral development and metabolic homeostasis [72]. In terms of limb development, FGFs signaling is associated with mesenchymal condensation, chondrogenic differentiation and hypertrophy, mineral homeostasis and bone formation [73]. There are four members of FGF receptors (FGFR1–FGFR4) family with diverse FGF-binding capacities and at least 22 FGF ligands that can be subdivided into seven groups [74–76]. The FGF receptor (FGFR) commonly is composed of an intracellular receptor tyrosine kinase domain, a hydrophobic transmembrane region and extracellular ligand-binding domains [77]. In the presence of FGF or other ligands, FGFR kinases are released from autoinhibition and auto-phosphorylated, whose phosphorylated tyrosine residues serve as attachment sites for recruiting interacting proteins [78]. The downstream signaling pathways such as phosphoinositide 3-kinase/Akt (PI3K–AKT) and protein kinase C pathways can be activated and transduce information into nucleus [79]. In general, FGFR2, FGF2, FGF8, FGF9, and FGF18 have been reported to involve in chondrogenic differentiation [80, 81]. FGFR2 is an early marker of chondrogenesis, whose expression pattern is restrained in mesenchymal condensation region before occurrence of chondroprogenitor [81, 82]. A gain-of-function mutation (S252W) of FGFR2 in mice results in dwindling proliferation BMSCs, a decline in BMSC chondrogenic differentiation via inhibiting mineralization [83], and changes in both Wnt signals and MAPK expression during human synovium-derived stem cell chondrogenic differentiation [84]. Solchaga et al. illustrated that FGF-2 functioned in the regulation of chondrogenic differentiation through MAPK and Wnt signaling via DUSP 4/6 and Fzd7, respectively [85]. In addition to FGF-2, FGFR1 could cooperate with β-catenin and alter the lineage commitment of MSCs into chondrocytes [86]. In combination with transforming growth factor-beta (TGF-β), FGF9 and FGF18 stimulated early chondrogenic differentiation by shifting the chondrogenic program earlier [87].

## Cytokines of chondrogenic differentiation in MSCs

### Transcription factor—Sox9

Sox9, sex determining region Y-box 9 [88], pertains to the SRY-related high-mobility group (HMG) box (Sox) family, whose family proteins are a conserved group of transcriptional regulators [89]. The fate and terminal differentiation of chondrocyte are regulated by Sox9 via its accurate spatial and temporal expression pattern [89–91]. Sox9 can be detected in chondroprogenitors and mature chondrocytes, but not in hypertrophic chondrocytes, and Sox9 directly upregulates genes specifically expressed in precartilaginous condensation [92]. Heterozygous mutations of Sox9 in human were first ascertained as the causative factor of campomelic dysplasia, a skeletal deformity with disorder of sexual development and genital ambiguities [93]. Mice with heterozygous Sox9 mutant can be perinatal mortality and presented palatoschisis and crookedness of bony structures originated from cartilage precursors [94]. As a part of the SoxE family, Sox9 possesses a distinct dimerization domain located proximally to the HMG box, which can interact with the enhancers and promoters of the aggrecan gene (Acan) and type-II collagen gene (Col2a1) [95–99], and a unique transactivation domain [100]. Sox9 with downstream transcription factors of SoxD family—Sox5 and Sox6 can work as chondrogenic Sox Trio [91]. Unlike Sox9, Sox5 and Sox6 are similar to one another in structure and function [101]. Sox5-deleted or Sox6-deleted mice develop modest skeletal disorders, yet deletion of these two genes could suffer serious cartilage primordia [91]. Zhou et al. have reported that the strong transcriptional activity of the 48 bp minimal enhancer of *Col2a1* gene appears just when Sox9, Sox5, and Sox6 (Sox5/6) bind together to the sites in the enhancer [102]. In addition to inducing overexpression of Col2a1 and Acan, Sox Trio inhibits hypertrophic gene expression and collagen type X deposition [103].

### Cartilage matrix protein—Col2a1 and aggrecan

The collagen α1(II) gene, located at 12q13, is a key element for production of alpha-1 type II collagen (Col2a1) [104], which is a main extracellular matrix protein of cartilage. Mutations in Col2a1 can result in spondyloepiphyseal dysplasia and type II achondrogenesis. The former is an autosomal dominant disease characterized by limb and trunk shortness, pulmonary hypoplasia, abdominal enlargement with polyuria and edema [105, 106]. In chondrogenic differentiation, expression profiles of Col2a1 are similar to the expressions of Sox9 in all chondroprogenitor cells in mouse and chick embryonic development [107–109]. There is a 48-bp gens in intron 1 sequence of *Col2a1* binding with Sox9, which is required for cartilage-specific expression [98, 102]. Besides SOX family, endogenous Hey-1 and Hes-1 can bind to N-box domains of the first intron of *Col2a1*, which is adjacent to the Sox9 enhancer binding site [110]. The response element of Runx1 has been identified in the 5-flanking regions of the Col2a1 promoter [111]. Transcription factors (Kruppel-like factor-4 and AT-rich interactive domain 5B) could combine with the E1 enhancer element in *Col2a1* and regulate its expression [112]. Aggrecan (Acan) is a large chondroitin sulfate proteoglycan consisting of a polypeptide backbone covalently attached to one or more glycosaminoglycan chains in growth plate cartilage [113, 114]. Four enhancer elements were identified in the aggrecan gene, two of which (− 80 and − 62) showed individual chondrocyte developmental stage specificity. The other enhancers specific to chondrogenesis, + 28 and − 30, were not associated with chondrocyte type [95]. Additionally, transcription factor Sox9 binds to the first enhancer of the aggrecan gene with or without Sox5 and Sox6 [115, 116]. In addition to Sox9, PAX1/9-binding site partly overlaps with a Sox9-binding site and exhibits as a weak transactivator [117].

## Non-coding RNA in chondrogenic differentiation

MiR-140 is involved in cartilage homeostasis and osteoarthritis development. miR-140-5p and miR-140-3p have been identified that are products of RNA Dicer excision at the 3′ and 5′ tail of the pre-miRNA, respectively [118]. This miRNA can bind to Sox9 directly or act on its upstream genes, such as RALA and SMAD3 [118–120]. Recent studies have confirmed the bioactivity of exosomes carrying miR-140 in chondrogenic differentiation of MSCs [121, 122]. MiR-23a and b are isoforms of miR-23 [123]. The former regulated by lncRNA SNHG5 decreases the expression of SOX6/SOX5 and inhibits chondrogenic differentiation, whereas miR-23a-3p silencing attenuates the differentiation effect of BMSCs [124, 125]. The latter induces differentiation into chondrocytes of hMSCs through the downregulation of protein kinase A signaling [126]. Recently, it was reported that FGF2 expression is negatively regulated by miR-23c, thereby affecting chondrogenic differentiation [127].

LncRNA DANCR, known as an anti-differentiation ncRNA, not only binds to protein-coding genes, but also regulates miRNAs, such as miR-1305 and miR-1275 in chondrogenesis [128–132]. Using CRISPR activation (CRISPRa) technology, DANCR improved adipose-derived stem cell chondrogenic differentiation by inhibiting miR-203a and miR-214 [133]. The lncRNA UCA1 has been proved to be relevant to several human cancers. The expression of UCA1 increased markedly along with chondrocyte differentiation [134]. UCA1 combined with miR-145-5p/SMAD5 and miR-124-3p/SMAD4 can regulate chondrogenic differentiation of MSCs [135].

According to the bioinformatics analysis of hybridization arrays, circRNAs associated with chondrogenic differentiation are derived from similar precursor genes, such as *FKBP5*, *ZEB1*, or *SMYD3* [136]. However, no experimental studies have reported the specific role of circRNAs in the cartilage lineage differentiation of stem cells [137].

## Biophysical factors in chondrogenic differentiation

Early stage of chondrogenic differentiation is enhanced and the expression of chondrogenic markers Col2a1, Sox9, and Acan are increased when MSCs are cultured under low oxygen tension [138, 139]. Besides the hypoxia factor, there are several mechanical cues in cartilage formation [140]. Fluid shear stress (ΔSS) is a potent regulator of chondrogenic differentiation, which is comparable to TGF-β1 induction [141, 142]. Hydrostatic pressure has an anabolic effect on MSCs chondrogenic differentiation, and the loading capacity and time, longitude of chondrogenic preprocessing prior to pressurizing, also have an effect on chondrogenic differentiation [143]. Lineage determination of MSCs is susceptible to material stiffness, which regulates this development through transforming growth factor beta (TGF-β) signaling pathway [144]. MSCs on softer substrates are prone to differentiation into chondrogenic lineage [145].

## Chondrogenic differentiation of malignant tumors

Cancer stem cells (CSCs), also known as tumor-initiating cells, are a small fraction of cells inside tumor tissues. They can self-renew and differentiate and are responsible for relapses as well as resistance to chemoradiotherapy [146, 147]. The ability of cancer cells to switch from non-CSCs to CSCs and vice versa is called phenotypic plasticity [148]. CSC plasticity takes determination to malignancy population dynamic and promotes cancer cellular progression [149–151]. Therefore, many researchers have exploited the plasticity of CSCs as a promising therapeutic target. The differentiation therapy implies that CSCs could be induced into differentiate into matured cells, and also converted into non-stem cells that were sensitive to traditional anticancer reagents [152]. The mesenchymal differentiation capacities of CSCs are involved in nervous system neoplasms [153, 154], bone sarcoma [155, 156], and prostate and cervical cancers [12, 157]. Polyploid giant cancer cells (PGCCs) induced by cobalt chloride have the properties of CSC characteristics and a single PGCC can form tumor in nude mice. When cultured in adipogenic, osteogenic and chondrogenic induction medium, PGCCs could differentiate into adipocytes, osteocytes and chondrocytes, respectively [158].

### Potential mechanism of CSCs inducible differentiation

Mesenchymal phenotype is a determinant for differentiation of CSC. Wilms tumors (WTs) are genetically heterogeneous kidney tumors. Five WT cell lines expressed MSC-specific surface proteins and had the ability to differentiation into adipogenic, chondrogenic, osteogenic and myogenic lineage [159]. ALDH + malignant phyllodes tumor cells could also be induced to differentiate into chondrocytes. CD133 + CSCs from osteosarcoma and Ewing’s sarcoma displayed the potency to differentiate into mesenchymal lineages, such as osteoblasts and chondrocytes [155, 156]. For epithelial-derived neoplasms, the transition between the epithelium and mesenchyme is a biological process called as epithelial–mesenchymal transition (EMT), in which epithelial cells obtain mesenchymal cell phenotypes and are related to mesenchymal differentiation [160]. Epithelial cells transduced with TGF-β1 retrovirus had the functional resemblance to MSCs, including a comparable antigenic phenotype (positive for CD44), the ability to lineage commitment (positive for Alcian blue of chondrocytes) [161]. It has been reported that the growth factor family (GF) regulated mesenchymal tri-lineage differentiation. Epidermal growth factor receptor (EGFR) inhibitors can induce the mesenchymal–epithelial transition and impact on EGF-induced EGFR signaling in breast CSC mesenchymal differentiation [162]. Transcriptional coactivator with PDZ-binding motif (TAZ) interacts with the transcription factor TEA domain family members (TEAD), which plays pivotal roles in EMT and cell growth. TAZ overexpression increases the expression of mesenchymal marker CD44 and the differentiation capability towards osteoblastic and chondrogenic lineage in glioma stem cells and murine neural stem cells [163].

Verhaak et al. described a robust gene expression-based molecular classification of GBM involved in proneural, neural, classical, and mesenchymal subtypes [164]. Analysis of The Cancer Genome Atlas (TCGA) demonstrated that RTVP-1 overexpressed in GBM which expressed mesenchymal phenotype, and silencing of RTVP-1 abrogated the chondrogenic differentiation of GBM cells in response to specific induction media. RTVP-1 promoter could bind C/EBP β, a master transcription factors that regulated mesenchymal transformation of GBM [165], and C/EBP β promoter activity could be suppressed by binding with Sox9 [166]. C/EBP β-expressing cells terminated in further increase of mesenchymal gene expression and obtained mesenchymal properties [167]. In gastric signet ring cell adenoma cancer, dysregulation of EMT-associated molecules and differentiation towards chondrocytes also occurred in KATO-III cell line (a cell line of gastric carcinoma) under the induction media [168].

The chondrogenic induction medium components in CSCs are same as those in MSCs, and include insulin-transferrin-selenium (ITS), ascorbate, TGF-β and dexamethasone. Exogenous TGF-β can stimulate TGF-β pathway, thereby accelerate the chondrogenic differentiation. Physiological levels of dexamethasone play a positive regulatory role in cartilage formation by directly interacting with the TGF-β signaling molecule, SMAD3 [169]. ITS is a nutritional supplement involved in glucose and proline metabolism during collagen synthesis. Ascorbate is a requisite cofactor in the production of collagen II and sulfated glycosaminoglycan [170]. The molecular mechanism of chondrogenic differentiation of CSCs may be similar to that of MSCs. Resveratrol inhibited MMP-induced chondrogenic differentiation via the p38 kinase and JNK pathways in chondrosarcoma cells [171]. The activation of p38/ERK/JNK pathways in the presence of TGF-β1 facilitated the expression of Sox9, collagen II and aggrecan [172]. MiR-200b-3p mediated chondrogenic differentiations of quercetin-induced pancreatic ductal adenocarcinoma CSCs by inhibiting Notch and activating Numb [173]. Sox9, Col2a1 and Acan was increased when transcription factor of notch pathway RBP-JK was restrained by DNA methyltransferase 3b [174].

### Differentiation therapy of CSCs

Retinoic acid (RA) is a well-known modulator in skeleton development, of which functions through a class of nuclear hormone receptors, the retinoic acid receptors (RARs) and retinoid-X-receptors (RXRs), to regulate gene transcription [175]. RA has successfully used in the differentiation therapy of acute promyelocytic leukemia [176]. RA acts on the RARA moiety of PML-RARA fusion protein that will be degraded, and contributes leukemia cells to terminal differentiation and apoptosis [177]. RA inhibited the condensation and proliferation and dysregulated Sox9 and Col2a1 in a dose-dependent manner through inhibition of Shh/Gli3 pathway [178]. Subtype-specific RAR agonists as well as RA had a strong inhibitory effect on chondrogenic differentiation with decreased the expression levels of Col2a1 and Sox9 [179]. Palovarotene (PVO), a RAR selective agonist, attenuates overactivated BMP signaling and restored aberrant chondrogenic fate determination of osteochondroma cells [180].

Traditional Chinese medicine has been widely accepted as an alternative treatment for cancer [173, 181]. In glioblastoma stem cells, the expression levels of proteins associated with differentiation, such as glial fibrillary acidic protein (GFAP), Notch1 and Shh, were increased by β-elemene in vitro and in vivo [182, 183]. Antagonist of CXCR4 (PRX177561) could regulate the tumor microenvironment, decrease the expression of CSC markers Sox2, Twist, Nanog, and overexpress GFAP in glioma [184]. Treatment of CD133 + CSCs derived from human GBMs with BMPs decreased cell proliferation in vitro and differentiated into astrocytes [185]. As a typical potassium ionophore antibiotic, Salinomycin improves epithelial differentiation ability of breast CSCs by means of eliminating cancer cells from CSC features and blocking cell cycle [186]. The inhibitor of HDACs (Quisinostat) stimulates DOX-mediated cytotoxicity in breast CSCs as well as non-CSCs from basal-like, mesenchymal-like, and luminal-like breast cancer [187]. In hepatocellular carcinoma, DW14800, a novel inhibitor of protein arginine methyltransferase 5 (PRMT5), promotes the differentiation of CSCs by downregulating the expression of HNF4α and enhancing methylation of H4R3me2s [188]. Metformin, a well-known anti-diabetic drug, can decrease CSC marker expression (CD44 and Sox2) and increase the expression of differentiation markers (Kruppel-like factor 4 and MUC5AC) in gastric CSCs [189]. IDH1 and IDH2 mutations are frequent in many solid cancers, and the enzymes encoded by these mutation are endowed with new function that facilitate the accumulation of the oncometabolite D-2-hydroxyglutarate (D-2HG), which has significant impacts on epigenetic regulation, differentiation degree, and metabolic patterns [190]. The differentiation inducers targeted mutant isocitrate dehydrogenase IDH1 and IDH2 have been granted for clinic trials. Ivosidenib, an IDH1 inhibitor, showed improved progression-free survival versus placebo in the phase III clinical trial of patients with advanced cholangiocarcinoma [191]. In advanced glioma, patients treated with ivosidenib were associated with a favorable safety profile, prolonged progression-free survival [192].

## Conclusion and perspective

Chondrogenic differentiation is a multifactorial and multistep process. Mesenchymal differentiation into chondrocytes is regulated by concurrent signaling pathways, among which the TGF-β pathway is the principal and the earliest signal in chondrogenic condensation. Engineered TGF-β superfamily ligands have been produced and tested for in vitro cartilage formation and used for commercial purposes. As transcriptomic and bioinformatics technologies have been developed, many of the identified ncRNAs have been validated for chondrogenic differentiation. LncRNA DANCR could improve mesenchymal differentiation to cartilage lineage by miRNAs (such as miR-203a and miR-214). CSCs, a small fraction of cancer cells with tumorigenesis, have ability to differentiate into non-CSCs because of phenotypic plasticity, which can be the basis for differentiation therapy. Retinoic acid (RA) has been accepted as the first-line treatment in acute promyelocytic leukemia. Retinoid signaling was also informed to play a negative role in chondrogenic differentiation. Therefore, it may be possible that retinoid antagonists directly transform malignant neoplasm into benign cartilage tissue. It has been reported that CSCs can differentiate along their lineage or undergo mesenchymal differentiation through EMT. CSCs could be induced to differentiate into adipogenic, osteogenic and chondrogenic cells in more than 10 kinds of malignant tumors. However, these studies just describe this differentiation phenomenon rather than clearly elucidate the mechanism underlying chondrogenic differentiation of CSCs. Understanding the molecular mechanism of induced differentiation of CSCs may be contribute to developing new drugs for cancer treatment.

## Data Availability

The authors declare that all data supporting the findings of this study are available within the article or contact the corresponding author upon reasonable request.

## Abbreviations

MSC: Mesenchymal stem cell
Sox: Sex determining region Y-box
CSC: Cancer stem cell
ECM: Extracellular matrix
TGF-β: Transforming growth factor-β
GDF: Growth differentiation factor
MAPK: Mitogen-activated protein kinase
TAK1: TGF-β-activated kinase 1
BMSC: Bone marrow stromal cell
BMP: Bone morphogenetic protein
Col2a1: Type-II collagen gene
Hh: Hedgehog
Smo: Smoothened
Ptch: Patched
Shh: Sonic hedgehog
Dhh: Desert hedgehog
Ihh: Indian hedgehog
NICD: Notch intracellular domain
Hes: Hairy and enhancer of split
Hey: Hes-related with YRPW motif
Fzd: Frizzled
GSK-3β: Glycogen synthase kinase 3
FGF: Fibroblast growth factor
FGFR: FGF receptor
HMG: High-mobility group
Acan: Aggrecan
PGCCs: Polyploid giant cancer cells
RA: Retinoic acid
RAR: Retinoic acid receptor
ITS: Insulin-transferrin-selenium
GFAP: Glial fibrillary acidic protein
GBM: Glioblastoma
ATO: Arsenic trioxide
IDH: Isocitrate dehydrogenase
HDAC: Histone deacetylase
EMT: Epithelial–mesenchymal transition
